# Bedaquiline-containing regimens in patients with pulmonary multidrug-resistant tuberculosis in China: focus on the safety

**DOI:** 10.1186/s40249-021-00819-2

**Published:** 2021-03-19

**Authors:** Jing-Tao Gao, Juan Du, Gui-Hui Wu, Yi Pei, Meng-Qiu Gao, Leonardo Martinez, Lin Fan, Wei Chen, Li Xie, Yu Chen, Hua Wang, Long Jin, Guo-Bao Li, Pei-Lan Zong, Yu Xiong, Qian-Hong Wu, Ming-Wu Li, Xiao-Feng Yan, Yan-Fang Miao, Qing-Shan Cai, Xin-Jie Li, Da-Peng Bai, Shu-Jun Geng, Guo-Li Yang, Pei-Jun Tang, Yi Zeng, Xiao-Hong Chen, Tong-Xia Li, Cui Cai, Yun Zhou, Ma Zhuo, Jian-Yun Wang, Wen-Long Guan, Lin Xu, Ji-Chan Shi, Wei Shu, Li-Li Cheng, Fei Teng, Yu-Jia Ning, Shi-Heng Xie, Yu-Xian Sun, Li-Jie Zhang, Yu-Hong Liu

**Affiliations:** 1 Clinical Center on TB, Beijing Chest Hospital, Capital Medical University/Beijing Tuberculosis and Thoracic Tumor Research Institute, No 9, Beiguan Street, Tongzhou District, Beijing, 101149 People’s Republic of China; 2 Department of Tuberculosis, Wuhan Pulmonary Hospital, Wuhan, People’s Republic of China; 3 Department of Tuberculosis, Chengdu Public Health Clinical Center, Chengdu, People’s Republic of China; 4 Department of Tuberculosis, Changsha Central Hospital, Changsha, People’s Republic of China; 5 Department of Tuberculosis, Beijing Chest Hospital, Capital Medical University/Beijing Tuberculosis and Thoracic Tumor Research Institute, Beijing, People’s Republic of China; 6 Division of Infectious Diseases and Geographic Medicine, School of Medicine, Stanford University, Palo Alto, CA USA; 7 Department of Tuberculosis, Shanghai Pulmonary Hospital, Shanghai, People’s Republic of China; 8 Department of Tuberculosis, Shenyang Chest Hospital, Shenyang, People’s Republic of China; 9 Department of Tuberculosis, The Sixth People’s Hospital of Zhengzhou, Zhengzhou, People’s Republic of China; 10 Department of Tuberculosis, Anhui Chest Hospital, Hefei, People’s Republic of China; 11 Department of Tuberculosis, Infectious Diseases Hospital Heilongjiang Province, Harbin, People’s Republic of China; 12 Department of Tuberculosis, The Third People’s Hospital of Shenzhen, Shenzhen, People’s Republic of China; 13 Department of Tuberculosis, Jiangxi Chest (Third People) Hospital, Nanchang, People’s Republic of China; 14 Department of Tuberculosis, Shandong Provincial Chest Hospital, Jinan, People’s Republic of China; 15 Department of Tuberculosis, Shanxi Provincial Tuberculosis Institute, Xi’an, People’s Republic of China; 16 Department of Tuberculosis, Kunming Third People’s Hospital, Kunming, People’s Republic of China; 17 Department of Tuberculosis, Chongqing Public Health Medical Center, Chongqing, People’s Republic of China; 18 Department of Tuberculosis, The Fourth People’s Hospital of Taiyuan, Taiyuan, People’s Republic of China; 19 Department of Tuberculosis, Hangzhou Red Cross Hospital, Hangzhou, People’s Republic of China; 20 Department of Tuberculosis, Guangzhou Chest Hospital, Guangzhou, People’s Republic of China; 21 Department of Tuberculosis, Tianjin Haihe Hospital, Tianjin, People’s Republic of China; 22 Department of Tuberculosis, Hebei Chest Hospital, Shijiazhuang, People’s Republic of China; 23 Department of Tuberculosis, Tuberculosis Hospital of Jilin Province, Changchun, People’s Republic of China; 24 Department of Tuberculosis, The Fifth People’s Hospital of Suzhou, Infectious Disease Hospital, Affiliated to Soochow University, Suzhou, People’s Republic of China; 25 Department of Tuberculosis, The Second Hospital of Nanjing, Nanjing, People’s Republic of China; 26 Department of Tuberculosis, Fuzhou Pulmonary Hospital of Fujian, Fuzhou, People’s Republic of China; 27 Department of Tuberculosis, Qingdao Chest Hospital, Qingdao, People’s Republic of China; 28 Department of Tuberculosis, Guiyang Public Health Clinical Center, Guiyang, People’s Republic of China; 29 Department of Tuberculosis, The Second Affiliated Hospital of Hainan Medical University, Haikou, People’s Republic of China; 30 Department of Tuberculosis, The Fourth People’s Hospital of QingHai Province, Xining, People’s Republic of China; 31 Department of Tuberculosis, Lanzhou Pulmonary Hospital, Lanzhou, People’s Republic of China; 32 Department of Tuberculosis, Chest Hospital of Xinjiang Uyghur Autonomous Region of the PRC, Urumchi, People’s Republic of China; 33 Department of Tuberculosis, The Fourth People’s Hospital of Ningxia Hui Autonomous Region, Yinchuan, People’s Republic of China; 34 Department of Tuberculosis, Wenzhou Central Hospital, Wenzhou, People’s Republic of China; 35 Beijing Innovation Alliance of TB Diagnosis and Treatment, Beijing, People’s Republic of China

**Keywords:** Tuberculosis, Multidrug-resistant, Bedaquiline, Safety, Surveillance program, China

## Abstract

**Background:**

World Health Organization recommends countries introducing new drug and short treatment regimen for drug resistant tuberculosis (DR-TB) should develop and implement a system for active pharmacovigilance that allows for detection, reporting and management of adverse events. The aim of the study is to evaluate the frequency and severity of adverse events (AEs) of bedaquiline-containing regimen in a cohort of Chinese patients with multidrug-resistant (MDR)/extensively drug-resistant (XDR)-TB based on active drug safety monitoring (aDSM) system of New Drug Introduction and Protection Program (NDIP).

**Methods:**

AEs were prospectively collected with demographic, bacteriological, radiological and clinical data from 54 sites throughout China at patient enrollment and during treatment between February, 2018 and December, 2019. This is an interim analysis including patients who are still on treatment and those that have completed treatment. A descriptive analysis was performed on the patients evaluated in the cohort.

**Results:**

By December 31, 2019, a total of 1162 patients received bedaquiline-containing anti-TB treatment. Overall, 1563 AEs were reported, 66.9% were classified as minor (Grade 1–2) and 33.1% as serious (Grade 3–5). The median duration of bedaquiline treatment was 167.0 [interquartile range (IQR): 75–169] days. 86 (7.4%) patients received 36-week prolonged treatment with bedaquiline. The incidence of AEs and serious AEs was 47.1% and 7.8%, respectively. The most frequently reported AEs were QT prolongation (24.7%) and hepatotoxicity (16.4%). There were 14 (1.2%) AEs leading to death. Out of patients with available corrected QT interval by Fridericia's formula (QTcF) data, 3.1% (32/1044) experienced a post-baseline QTcF ≥ 500 ms, and 15.7% (132/839) had at least one change of QTcF ≥ 60 ms from baseline. 49 (4.2%) patients had QT prolonged AEs leading to bedaquiline withdrawal. One hundred and ninety patients reported 361 AEs with hepatotoxicity ranking the second with high occurrence. Thirty-four patients reported 43 AEs of hepatic injury referred to bedaquiline, much lower than that referred to protionamide, pyrazinamide and para-aminosalicylic acid individually.

**Conclusions:**

Bedaquiline was generally well-tolerated with few safety concerns in this clinical patient population without any new safety signal identified. The mortality rate was generally low. These data inform significant positive effect to support the WHO recent recommendations for the wide use of bedaquiline.

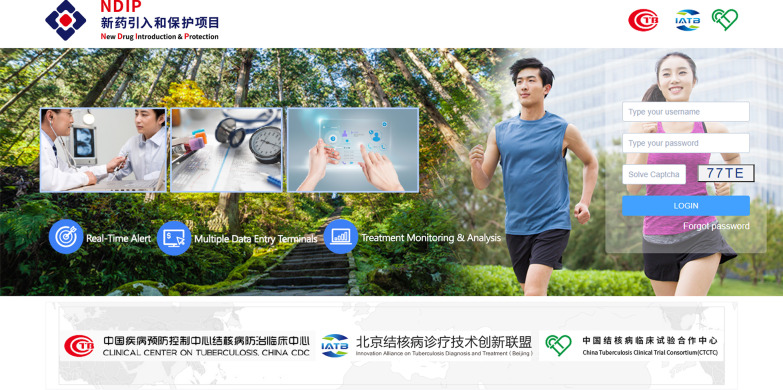

**Supplementary Information:**

The online version contains supplementary material available at 10.1186/s40249-021-00819-2.

## Background

Multidrug-resistant (MDR) and extensively drug-resistant (XDR) tuberculosis (TB) are critical threats to global health [1, 2]. It is newly reported approximately 361 000 new cases of MDR-TB emerged globally [1], and China accounted for 14% [1]. In 2019, 7.1% of new cases and 23% of previously treated cases were estimated rifampicin resistant TB (RR-TB)/MDR-TB in China. However, the average treatment success of MDR/RR-TB is 57% while it is only 39% of XDR-TB [1]. Novel effective and safe drugs are urgently needed to improve treatment outcomes of MDR/XDR-TB and prevent further drug resistance.

Previously considered a group D2 drug, bedaquiline now belongs to group A in the World Health Organization (WHO) guidelines [3]. Bedaquiline is now considered effective against MDR/XDR-TB, with a manageable toxicity profile [4–9]. Among the major safety concerns of bedaquiline is alterations in the QT interval [10], requiring regular electrocardiogram (ECG) monitoring.

Inappropriate antibiotic use including anti-TB drugs is a common phenomenon in some hospitals in China [11–13]. Regulation of antibiotic circulation, supply, and application is critical to prevent resistance among novel drugs. However, this is uncommon, potentially greatly compromising decades of drug development by the international community. On November 23, 2016, bedaquiline received conditional approval by the China National Medical Products Administration. At the time of its approval in China, bedaquiline utilization data for Chinese MDR-TB patients were very limited. The first access to bedaquiline was via a national program named New anti-TB Drugs Introduction and Protection Program (NDIP). It was set up under the umbrella of China-BMGF stage III project during 2016–2019 and was implemented by the Clinical Center on Tuberculosis of the Chinese Center for Disease Control and Prevention (China CDC) under the guidance and support of the National Health Commission of China and the Gates Foundation. The aim of NDIP is to establish effective mechanisms for ensuring the correct, appropriate and safe use as well as to prevent resistance to novel anti-TB drugs in China under surveillance. Bedaquiline is the first drug to test the new model and mechanism of NDIP.

The electronic pharmacovigilance system at NDIP is developed according to WHO recommendation to implement “active and systematic clinical and laboratory assessment of patients on treatment with new TB medicines, or novel MDR-TB regimens in order to detect and report potential or confirmed drug toxicities” [14–16].

We aimed to prospectively evaluate the frequency and severity of AEs in MDR/XDR-TB patients treated with bedaquiline-containing regimen in China. An interim finding was summarized of patients who completed or were still on treatment through the NDIP at the time of data collection.

## Methods

### Study participant enrollment

Eligible patients were enrolled to NDIP from 54 hospitals around China from February 2018 to December 2019 with China first approved new anti-TB drug, bedaquiline, donated by Global Drug Facility under active drug safety monitoring (aDSM) framework. Inclusion criteria were: (1) laboratory diagnosis of MDR/XDR-TB; (2) failure to respond to current MDR-TB regimens lacking bedaquiline; (3) ≥ 18 years of age; (4) no respiratory failure, cardiac failure, clinically significant arrhythmia, or corrected QT interval by Fridericia's formula (QTcF) < 450 ms. Exclusion criteria were: (1) allergy to bedaquiline; (2) participation in other clinical trials within the past three months; (3) pregnant or breast-feeding; (4) concomitant serious illness, including alanine aminotransferase/aspartate aminotransferase (ALT/AST) > 3 × upper limits of normal (ULN) or total bilirubin > 2 × ULN, creatinine clearance < 30 ml/min, haemoglobin ≤ 70 g/L and/or platelets < 50 × 10^9^/L at screening. (5) history of high-risk cardiac comorbidities (e.g., ventricular arrhythmia, myocardial infarction) with risk factors of QT prolongation: a. ECG at screening showing evident QT interval or QTcF ≥ 450 ms (an unscheduled visit was allowed for ECG reexamination during the screening period to re-evaluate patient eligibility); b. pathologic Q wave (Q wave > 40 ms or depth of Q wave > 0.4–0.5 mV); c. evidence of ventricular preexcitation (e.g., Wolff-Parkinson-White syndrome); d. ECG showed evidence of complete or clinically significant incomplete left bundle branch block or right bundle branch block; e. evidence of Grade II or III heart block; f. intraventricular conduction delay, QRS duration > 120 ms; g. bradycardia (sinus heart rate < 50 bpm); h. personal or family history of long QT syndrome; i. history of heart disease, symptomatic or asymptomatic arrhythmia (except for sinus arrhythmia); j. cardiogenic syncope; or k. have risk factors for developing torsades de pointes (TdP), such as heart failure, hypokalemia, or hypomagnesemia.

### Protocol training and data management

According to the NDIP protocol, medical professionals of selected TB specialized hospitals capable of MDR-TB diagnosis and treatment were well trained for patient enrolment, bedaquiline-containing regimen design, drug administration, treatment outcome and safety monitoring and evaluation. Standardized electronic case report form (eCRF) were filled in by trained doctors in each hospital and data was reviewed by an independent data monitoring committee of NDIP routinely. NDIP was approved by the ethics committee of each participating hospital. All patients enrolled provided written informed consent.

### Treatment regimen

According to WHO guidelines and NDIP protocol, local physicians developed individualized background regimens based on patients’ previous histories of anti-TB treatment and drug susceptibility testing (DST) results as well as drug tolerance. For patients with DST results, bedaquiline was used in combination with at least three background drugs to which their TB isolate was susceptible. For patients without definitive DST results, bedaquiline was used in combination with at least four drugs to which the isolate was likely to be susceptible based on treatment history and local epidemiology of drug resistance. Bedaquiline was administered at the recommended dose of 400 mg once a day for 14 days then at a dose of 200 mg three times per week for the remaining 22 weeks.

Background regimens consisted of the anti-TB drug formulations guided by DST, including moxifloxacin, levofloxacin, linezolid, clofazimine, amikacin, capreomycin, protionamide, cycloserine, pyrazinamide, ethambutol, para-aminosalicylic acid, high-dose isoniazid, meropenem and amoxicillin/clavulanate. To ensure patient adherence to outpatient treatment, patients were supervised by trained professional clinicians, who monitored patient treatment progress and provided medical and psychosocial support. Prolongations of QTcF more than 500 ms were investigated, and treatment was modified if the prolongation was considered to be drug related by the site investigators.

### Safety monitoring and evaluation

Patients were followed every 2 weeks for the first month and every 4 weeks thereafter. Information regarding demographic characteristics including age, sex, height, weight, clinical history, medication history, background regimens, laboratory test results, ECG and AEs, bacteriological, radiological findings were collected from the NDIP information monitoring system.

AEs in the NDIP CRF were coded using the Medical Dictionary for Regulatory Activities (MedDRA). Number and percentage of patients with AEs were summarized by MedDRA system organ class (SOC) and preferred term (PT). Number and proportion of patients with AEs were also summarized by severity (Grade 1, Grade 2, Grade 3, Grade 4 and Grade 5). Number and percentage of patients with AEs were summarized by causality (related, not related) with bedaquiline. Adverse events were judged as related with bedaquiline if the causality was related, probably related, possibly related, unable to determine or missing; judged as not-related with bedaquiline if the causality was unlikely related or as not-related.

AEs were graded according to the guidelines Division of AIDS for Grading the Severity of Adult and Pediatric Adverse Events, version 2.1 [17]. Serious AEs included death or a life-threatening event, hospitalization or prolongation of hospitalization, persistent or significant disability, or congenital anomaly. Serious AEs included Grade 3–5 AEs (Grade 3: serious; Grade 4: life threatening; Grade 5: death) [14, 17]. Minor AEs included those of Grade 1 (mild) and Grade 2 (moderate) [14, 17].

This paper reported the results of the interim analysis conducted on the data from February 24, 2018 up to December 31, 2019.

### Data analysis

A descriptive analysis was performed on the patients evaluated in the cohort using SAS 9.4 (SAS Institute Inc., Cary, NC, USA). Continuous variables with normal distribution were reported as mean ± standard deviation (SD) while non-normal distribution variables were reported as median [interquartile range (IQR)]. Categorical variables were summarized using numbers and percentages. The numbers of patients with absolute value ≥ 450 ms and ≥ 500 ms, and changes from baseline ≥ 30 ms and ≥ 60 ms were summarized for the QTcF results. Boxplots of QTcF for 24 weeks and the trend chart of QTcF changes from baseline to 24 weeks were plotted.

## Results

### Characteristics of the participants

Overall, 1162 eligible patients were enrolled into NDIP and treated with bedaquiline-containing regimens from 54 hospitals of 32 provinces nationwide between February 2018 and December 2019.

The characteristics of the patients is presented in Table 1. The median age was 36.0 (IQR: 28–50) years, and 70.0% of patients were male. Among the 1162 patients, 288 (24.8%) were diagnosed with XDR-TB, 492 (42.3%) were diagnosed with pre-XDR-TB, and 382 (32.9%) were diagnosed with MDR-TB. Most (79.9%) patients were previously treated, while 233 (20.1%) patients were treatment-naïve. Pulmonary TB was diagnosed in all the cases (100%), with 103 (8.8%) involvement of both pulmonary and extra pulmonary sites (66 pleural, 10 bone and joint, 15 lymph nodes, 7 central nervous system, 5 gastrointestinal). A total of 360 (31%) patients reported the use of concomitant medications. The most frequently used concomitant medications by anatomical therapeutic chemical (ATC) class II included biliary and liver therapy (*n* = 120, 10.3%), diabetic drugs (*n* = 100, 8.6%), all other therapeutic products (*n* = 89, 7.7%), vitamin supplements (*n* = 118, 10.2%), and unspecified herbal and traditional Chinese medicine (*n* = 44, 3.8%).

**Table 1:** Demographic and clinical characteristics of 1162 MDR/XDR tuberculosis patients in China

Characteristics	Patients (*n* = 1162)
Median age (IQR), years	36.0 (28, 50)
Sex, *n* (%)	
Male	813 (70.0)
Female	349 (30.0)
Height (cm), mean ± SD	169.3 ± 7.9
Weight (kg), mean ± SD	59.4 ± 12.2
Pattern of drug resistance, *n* (%)	
MDR-TB	382 (32.9)
Pre-XDR-TB	492 (42.3)
XDR-TB	288 (24.8)
Previous anti-TB therapy, *n* (%)	
New	233 (20.1)
Previously treated	929 (79.9)
Site of TB, *n* (%)	
Pulmonary TB	1162 (100)
Concomitant extra-pulmonary TB	103 (8.9)

IQR, interquartile range; SD, standard deviation; MDR, multidrug-resistant; XDR, extensively drug-resistant; TB, tuberculosis

The median duration of bedaquiline treatment in the cohort was 167.0 (IQR: 75–169) days. Eighty-six (7.4%) patients received 36-week prolonged treatment of bedaquiline. Among the 1162 patients, 99.3% of the patients had good adherence (≥ 80%) to bedaquiline. As of December 31, 2019, there were 619 (53.3%) patients who completed bedaquiline treatment, 52 (4.5%) patients with bedaquiline discontinued due to AEs and the remaining 491 (42.3%) continue to be on a bedaquiline-containing regimen.

### Background regimens

The most frequently used background drugs included linezolid (*n* = 1030, 88.6%), cycloserine (*n* = 962, 82.8%), clofazimine (*n* = 694, 59.7%), protionamide (*n* = 592, 50.9%), amikacin (*n* = 482, 41.5%), moxifloxacin (*n* = 467, 40.2%), para-aminosalicylic acid (*n* = 399, 34.3%), and pyrazinamide (*n* = 293, 25.2%) (Table 2). Clofazimine and moxifloxacin are the known QT prolonging drugs among them.

**Table 2:** Background regimens used in combination with bedaquiline

Drug	Patients (*n* = 1162),* n* (%)
Linezolid	1030 (88.6)
Cycloserine	962 (82.8)
Clofazimine	694 (59.7)
Protionamide	592 (50.9)
Amikacin	482 (41.5)
Moxifloxacin	467 (40.2)
Aminosalicylic acid	399 (34.3)
Pyrazinamide	293 (25.2)
Capreomycin	214 (18.4)
Levofloxacin	157 (13.5)
Ethambutol	132 (11.4)
Amoxicillin; clavulanate potassium	61 (5.2)
Pasiniazid	17 (1.5)
Isoniazid	14 (1.2)
Clarithromycin	8 (0.7)
Cilastatin; imipenem	1 (0.1)
Gatifloxacin	1 (0.1)
Meropenem	1 (0.1)
Rifabutin	1 (0.1)
Streptomycin	1 (0.1)
Terizidone	1 (0.1)

### Adverse events and adverse drug reactions

From February 24, 2018 to December 31, 2019, 547 (47.1%) patients reported 1563 AEs. The frequently reported AEs (≥ 2% of patients) were QT prolongation (*n* = 287, 24.7%), hepatotoxicity (*n* = 190, 16.4%), blood disorder (*n* = 64, 5.5%), nephrotoxicity (*n* = 53, 4.6%), electrolyte imbalance (*n* = 49, 4.2%), gastrointestinal disorder (*n* = 48, 4.1%), peripheral neuropathy (*n* = 48, 4.1%), ototoxicity (*n* = 31, 2.7%), vestibular disorder (*n* = 31, 2.7%), and optic neuritis (*n* = 28, 2.4%) (Table 3). Summary of AE by SOC and PT is presented in Additional file 1: Table S1. Grades of the 1563 AEs are categorized in Additional files 1 as well.

**Table 3:** Frequency of adverse events reported

Events	Patients (*n* = 1162),* n* (%)
Any AE	547 (47.1)
Bedaquiline-related AE	278 (23.9)
SAE	91 (7.8)
Bedaquiline-related SAE	45 (3.9)
AE leading to death	14 (1.2)
Bedaquiline-related AE leading to death	0
AE leading to bedaquiline withdrawal	52 (4.5)
QT prolongation leading to bedaquiline discontinuation	49 (4.2)
AE reported ≥ 2% of patients	
QT prolongation	287 (24.7)
Hepatotoxicity	190 (16.4)
Blood disorder	64 (5.5)
Nephrotoxicity	53 (4.6)
Electrolyte imbalance	49 (4.2)
Gastrointestinal disorder	48 (4.1)
Peripheral neuropathy	48 (4.1)
Ototoxicity	31 (2.7)
Vestibular disorder	31 (2.7)
Optic neuritis	28 (2.4)

AE, adverse event; SAE, serious adverse event

Adverse drug reactions (ADRs) are AEs with causality and AEs with missing causality were not reported as ADR in this study. As of the study cut-off date, 278 (23.9%) patients reported 516 ADRs (Table 3). Three most frequently reported ADRs (≥ 1.0% of patients) were QT prolongation (*n* = 243, 20.9%), hepatotoxicity (*n* = 34, 2.9%), and gastrointestinal disorder (*n* = 14, 1.2%). Summary of ADR by SOC, PT is presented in Additional file 1: Table S2.

### Serious adverse events and serious adverse drug reactions

Ninety-one (7.8%) patients experienced 151 serious AEs (SAEs). A summary of SAE by SOC, PT is presented in Additional file 1: Table S3. Forty-five (3.9%) patients experienced 68 serious ADRs (SADRs). Summary of SADR by SOC and PT is presented in Additional file 1: Table S4.

### Severity of adverse events and adverse drug reactions

Grade 1 and Grade 2 AEs were reported by 149 (12.8%) and 122 (10.5%) patients, respectively. Two hundred and nineteen (18.8%) patients experienced Grade 3 AEs among whom QT prolongation (12.4%) was reported in high incidence. Grade 4 and Grade 5 AEs were reported by 38 (3.3%) and 13 (1.1%) patients. As per AE reporting page and NDIP database, one patient with Grade 1 hepatotoxicity died suddenly and the outcome of the hepatotoxicity was written death but no Grade 5 AE was reported. Thus 14 subjects with outcome of death, while 13 subjects with Grade 5 AEs were reported. A summary of AEs by SOC, PT and severity is provided in Additional file 1: Table S5. The number of patients who experienced Grade 3 and Grade 4 ADRs was 148 (12.7%) and 12 (1.0%), respectively. No Grade 5 ADRs were reported. Summary of ADR by SOC, PT and severity is presented in Additional file 1: Table S6.

### Outcomes of adverse events and adverse drug reactions

AE outcomes were reported as improvement in 263 (22.6%) patients, no improvement in 95 (8.2%) patients, cured in 78 (6.7%) patients, recovery with sequelae in 5 (0.4%) patients, unknown in 41 (3.5%) patients, death in 14 (1.2%) patients and with sequelae in 5 (0.4%) patients. Summary of AEs by SOC, PT and outcome is presented in Additional file 1: Table S7. ADR outcomes were reported as improvement in 119 (10.2%) patients, cured in 67 (5.8%) patients, no improvement in 34 (2.9%) patients and unknown in 33 (2.8%) patients. There was no reported recovery with sequelae or death due to ADRs. Summary of ADR by SOC, PT and outcome is presented in Additional file 1: Table S8.

### AEs leading to death and leading to bedaquiline withdrawal

Fourteen (1.2%) patients experienced AEs leading to death. Summary of AEs leading to death by SOC and PT is presented in Additional file 1: Table S9. No patient experienced ADRs leading to death. Fifty-two (4.5%) patients experienced AEs leading to bedaquiline withdrawal. Out of 52 patients, 49 (4.2%) patients with QT prolonged AEs led to bedaquiline withdrawal, and Grade 3 and Grade 4 QT prolongation were reported by 45 and 4 patients, respectively.

### Hepatobiliary disorders

One hundred and ninety (16.4%) patients reported 361 AEs of hepatobiliary disorders including hepatotoxicity, hepatic function abnormal and hyperbilirubinemia. Grade 1 and Grade 2 AEs of hepatobiliary disorders were reported by 132 (69.5%) and 43 (22.6%) patients, respectively. The numbers of patients who experienced Grade 3 and Grade 4 AEs were 10 (5.3%) and 5(2.6%) accordingly. No Grade 5 AEs of hepatobiliary disorders were reported. Eighty-six patients reported 156 AEs with the causality of protionamide being related, probably related or possibly related, followed by para-aminosalicylic acid with 52 patients reported 88 AEs, pyrazinamide with 50 patients reported 88 AEs, and left the bedaquiline least with 34 patients reported 43 AEs of hepatobiliary disorders. Among the 34 patients with ADRs caused by bedaquiline, the numbers of patients with Grade 1 and Grade 2 ADRs were 22 (64.7%) and 6 (17.6%). Grade 3 and Grade 4 ADRs were reported by 1 (2.9%) and 5 (14.7%) patients. No Grade 5 ADRs referred to bedaquiline was reported.

### QT prolongation profiles

**Fig. 1:**
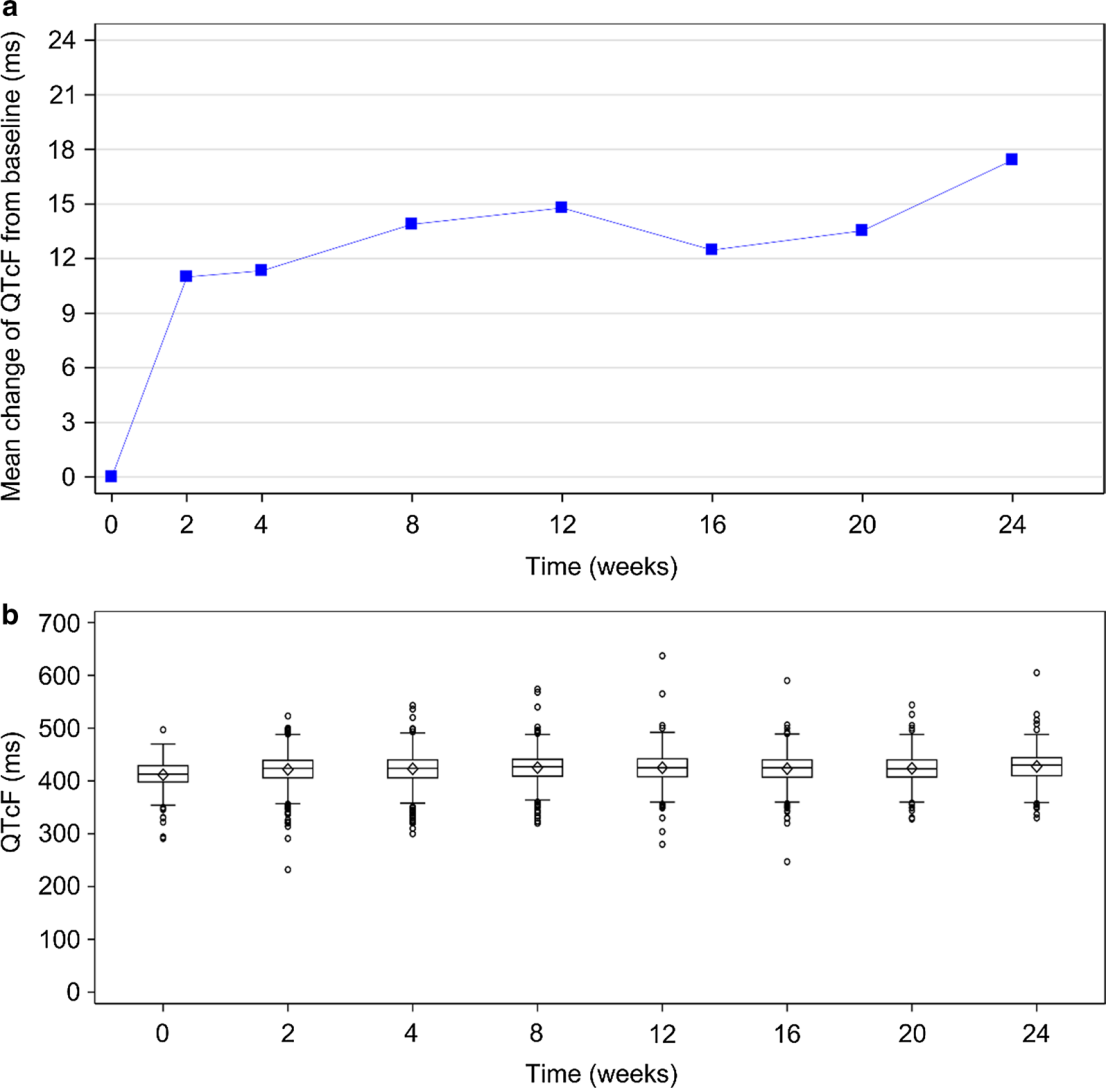
QTcF values monitoring at different time points during treatment with bedaquiline-containing regimen. **a** Overall trend chart of QTcF increment from baseline at different time points. **b** QTcF values at different time points during treatment. Boxes represented the median and IQR. QTcF, corrected QT interval by Fridericia's formula

At baseline, the median QTcF was 413 (IQR: 398–429) ms. No patient had a QTcF ≥ 500 ms at baseline. The median change in QTcF from baseline to week 24 was 16 (IQR: -3–35) ms. Among 1,044 (89.8%) patients with at least one QTcF value over follow-up, 424 (40.6%) demonstrated follow-up QTcF ≥ 450 ms; 32 (3.1%) patients had follow-up QTcF ≥ 500 ms. Among 839 (72.2%) patients with both baseline and at least one post-baseline value, 439 (52.3%) patients reported an increase of more than 30 ms from baseline. In addition, 132 (15.7%) patients reported an increase of more than 60 ms from baseline (Table 4). Fluctuations in QTcF intervals were generally stable from week 2 to 24 (Fig. 1).

**Table 4:** QTcF profiles of patients who received bedaquiline-containing regimen in the cohort

Variable	Patients (*n* = 1162),* n* (%)
Median QTcF at baseline (IQR), ms	413 (398, 429)
Median change in QTcF from baseline to week 24 (IQR), ms	16 (−3, 35)
Worsening QTcF from baseline to follow-up, *n* (%)	
*n*	1044
≥ 450 ms	424 (40.6)
≥ 500 ms	32 (3.1)
Change in QTcF from baseline, *n* (%)	
*n*	839
≥ 30 ms	439 (52.3)
≥ 60 ms	132 (15.7)

IQR, interquartile range; QTcF, corrected QT interval by Fridericia's formula

## Discussion

This large-scale, multi-center and prospective study aimed to evaluate the frequency and severity of AEs with novel anti-TB drug bedaquiline-containing regimen in China. Out of 1162 enrolled MDR/XDR-TB patients, 1563 AEs were reported from 547 (47.1%) patients. The first important contribution of the study presented the first step and efforts China has made in implementing aDSM in practice and scientific evidence has been actively collected nationwide. The second important finding of this study is trying to explore attribution of the AEs to a specific drug based on evidence-based profile. The results demonstrated that 278 patients have reported 516 ADRs. The AEs were referred to bedaquiline for 285 (24.5%) patients which is much higher than that at 11.1% from recent first global report [18], close and intensified safety monitoring acted on in NDIP. Thirdly, there is no new safety signals were uncovered of Chinese MDR/XDR-TB patients exposed to bedaquiline-containing regimen. Our results also demonstrated again that QT prolongation and hepatotoxicity are the most frequent adverse effects exposed to bedaquiline.

Drug adherence to bedaquiline was generally good as more than 80% of doses were taken of those prescribed, indicating the acceptability and tolerability of it. The concomitant medications for other comorbidities may also lead to the occurrence of AEs. In this study, approximately 10% of patients were using drugs for biliary and hepatic diseases as well as diabetes. Unfortunately, the NDIP database did not contain data about the exact concomitant drugs used by each patient, limiting our ability to analyze drug interactions among patients with both TB and other comorbidities.

ADR with hepatotoxicity was reported with higher frequency in patients with protionomide, followed by para- aminosalicylic acid, pyrazinamide and bedaquiline which could be explained by the number of patients with their concurrent use as background components with bedaquiline (see Table 2).

All reported AEs were expected and manageable with symptomatic treatments. Considering previous reports on the safety of bedaquiline [4, 5, 7, 18–22], patient QTcF profiles should continue to be monitored. Our results on acceptable safety profile of bedaquiline supports previous data published by a multi-centre study conducted in 15 countries [5], a phase 2b TMC207-C208 study [19], and a phase 2 TMC207-C209 study [20]. QT prolongation is associated with increased risk of TdP which may cause sudden cardiac death if severe. The frequent use of clofazimine (59.7%) and moxifloxacin (40.2%) as part of the background regimen for many patients in the present study may contribute to QT prolongation due to the WHO guideline update and access of these off-label drugs in China recent years, while only 21.6% of patients co-administrating clofazimine and 2.0% moxifloxacin in C209 Chinese subgroup during 24-week bedaquiline-containing regimen treatment. Notably, according to the first global aDSM report [18], overall 2.6% patients experienced a QTcF prolongation ≥ 500 ms and 8 patients reported serious cardiological AEs among whom 4 attributed to bedaquiline, 2 due to clofazimine, 1 due to moxifloxacin and PAS, while 1 due to a non-TB drug. Nevertheless, under the strict monitoring of ECG by NDIP, QT prolongation was infrequent (3.4%). As for the detailed QTcF profile, 15.7% of patients had change in QTcF ≥ 60 ms from baseline, but only 3.1% of patients had post-baseline QTcF ≥ 500 ms. This was within the acceptable range.

Regarding bedaquiline discontinuation due to AEs (4.5%) and QTcF prolongation (4.2%), it was slightly higher than what was reported in other studies. In the largest systematic and critical analysis report with published data including clinical trial and observational studies in 2017, the drug was discontinued in 3.4% and 0.6% of patients due to AEs and QTcF prolongation, respectively. And in another larger study conducted by TBNet [23], out of 1044 bedaquiline-treated patients, this drug had to be stopped in 8 cases following QTcF prolongation (0.77%, 95% *CI:* 0.04–1.57%). This was similar to what was observed in conditional access program of bedaquiline conducted in India [24], with permanent withdrawal of bedaquiline in 4% (27 of 620 patients) patients for AEs, including prolonged QTcF (2.9%, 18 of 620 patients). Recent published meta analysis [25] showed that bedaquiline, together with fluoroquinolones, clofazimine had the lowest incidence of AEs leading to permanent drug discontinuation, whereas second-line injectable drugs, aminosalicylic acid, and linezolid had the highest incidence.

Mortality occurred in approximately 1% of our study cohort, none directly related to bedaquiline use. This rate is substantially lower than those from prior studies. For example, the mortality rate was 13%, 7%, and 12% in the TMC207–C208 study [19], the TMC207–C209 study [20], and a pooled analysis of five observational studies [22]. Bedaquiline received conditional regulatory approval in the initial stages of drug evaluation due to an apparent increased risk of death in a preliminary clinical trial. However, an abundant of subsequent studies have shown substantially reduced mortality among those taking bedaquiline [8, 26]. Nevertheless, pharmacovigilance is essential and we await the coming results of several randomized phase III clinical trials and observational studies to bring more safety evidence. Reasons for the low incidence of death may be attributed to the cautious management and strict patient monitoring in our study. Further research is needed to understand differences in mortality among patients taking bedaquiline in our settings.

There are limitations to this study. First, there was no control group of comparable patients who did not take bedaquiline. Second, patients had a heterogeneous exposure to bedaquiline. A proportion of patients completed bedaquiline treatment while the remaining were still on treatment as of study cut-off date. Different exposure may have an impact on the occurrence of AEs. Third, causality assessment of bedaquiline associated adverse events is challenging because patients often have co-morbid conditions which may be implicated in the adverse event. Moreover, patients are always taking several background medicines for treatment of MDR/XDR-TB. Many of these have overlap effects on adverse reaction with bedaquiline. Fourth, higher frequency of QTcF prolongation than what was reported in other studies may be explained by data collection including that non-anti-TB drugs data was not available; data on concomitant use of other QT-prolonging drugs (moxifloxacin, clofazimine and clarithromycin) was not analyzed; issues with either manual (differences between people reading) or automated readings (if different machines used) may affect. Fifth, we only analyzed changes in QT interval from baseline to 24 weeks of treatment, however, bedaquiline was extended to 36 weeks in 7.4% of patients. Follow-up time may be inadequate to observe QT interval changes. In addition, since bedaquiline has a long half-life of 5.5 months [27], specific AEs may occur after ceasing bedaquiline use. Future studies are necessary to determine the optimal use of bedaquiline and obtain an overall, accurate safety evaluation.

## Conclusions

The study results affirmed the relative safety of bedaquiline-containing regimen in patient cohort with MDR/XDR-TB in China which informs significant positive effect to support the WHO recent recommendations for the wide use of bedaquiline. Furthermore, the aDSM system established and applied in NDIP provided standardized, regular, close and active recording and reporting model with common protocol which is feasible and valuable for safety monitoring and evaluation of coming new drugs and regimens.

## Supplementary Information

**Additional file 1**: **Table S1.** Summary of AE by System Organ Class and Preferred Term. **Table S2.** Summary of ADR by System Organ Class, Preferred Term. **Table S3.** Summary of SAE by System Organ Class, Preferred Term. **Table S4.** Summary of SADR by System Organ Class and Preferred Term. **Table S5.** Summary of AE by System Organ Class, Preferred Term and Severity. **Table S6.** Summary of ADR by System Organ Class, Preferred Term and Severity. **Table S7.** Summary of AE by System Organ Class, Preferred Term and Outcomes. **Table S8.** Summary of ADR by System Organ Class, Preferred Term and Outcomes. **Table S9.** Summary of AE Leading to Death by System Organ Class and Preferred Term.

## Data Availability

The datasets generated during and/or analyzed during the current study are available from the corresponding author on reasonable request.

## Abbreviations

aDSM: Active drug safety monitoring
AEs: Adverse events
ALT: Alanine aminotransferase
AST: Aspartate aminotransferase
China CDC: Chinese Center for Disease Control and Prevention
DR-TB: Drug resistant tuberculosis
DST: Drug susceptibility testing
ECG: Electrocardiogram
eCRF: Electronic case report form
IQR: Interquartile range
MDR-TB: Multidrug-resistant tuberculosis
MedDRA: Medical Dictionary for Regulatory Activities
NDIP: New Drug Introduction and Protection Program
PT: Preferred term
QTcF: Corrected QT interval by Fridericia's formula
SOC: System organ class
TB: Tuberculosis
TdP: Torsades de pointes
ULN: Upper limits of normal
WHO: World Health Organization
XDR-TB: Extensively drug-resistant tuberculosis
