# Supplementary material for: Bedaquiline-containing regimens in patients with pulmonary multidrug-resistant tuberculosis in China: focus on the safety
Source: Infect Dis Poverty. 2021 Mar 19;10:32. doi: 10.1186/s40249-021-00819-2 (PMC7977294; doi:10.1186/s40249-021-00819-2)
Supplement: Supplementary file 1 — Additional file 1: Table S1. Summary of AE by System Organ Class and Preferred Term. Table S2. Summary of ADR by System Organ Class, Preferred Term. Table S3. Summary of SAE by System Organ Class, Preferred Term. Table S4. Summary of SADR by System Organ Class and Preferred Term. Table S5. Summary of AE by System Organ Class, Preferred Term and Severity. Table S6. Summary of ADR by System Organ Class, Preferred Term and Severity. Table S7. Summary of AE by System Organ Class, Preferred Term and Outcomes. Table S8. Summary of ADR by System Organ Class, Preferred Term and Outcomes. Table S9. Summary of AE Leading to Death by System Organ Class and Preferred Term. [file 40249_2021_819_MOESM1_ESM.docx]

Supplement tables

| Table S1: Summary of AE by System Organ Class and Preferred Term | |
| --- | --- |
| System Organ Class | Bedaquiline (N=1162) |
| Preferred Term | NE n (%) |
| Number of Subjects with at least one AE | 1563 547 (47.1) |
|  | |
| Investigations | 576 290 (25.0) |
| Electrocardiogram QT prolonged | 569 287 (24.7) |
| Heart rate decreased | 2 2 (0.2) |
| Blood urine present | 1 1 (0.1) |
| Electrocardiogram ST-T change | 1 1 (0.1) |
| Electrocardiogram T wave abnormal | 1 1 (0.1) |
| Platelet count decreased | 1 1 (0.1) |
| QRS axis abnormal | 1 1 (0.1) |
|  | |
| Hepatobiliary disorders | 361 191 (16.4) |
| Hepatotoxicity | 355 190 (16.4) |
| Hepatic function abnormal | 4 1 (0.1) |
| Hyperbilirubinaemia | 2 1 (0.1) |
|  | |
| Nervous system disorders | 117 94 (8.1) |
| Neuropathy peripheral | 59 48 (4.1) |
| Optic neuritis | 33 28 (2.4) |
| Headache | 6 6 (0.5) |
| Dizziness | 6 5 (0.4) |
| Syncope | 3 3 (0.3) |
| Hypoaesthesia | 2 2 (0.2) |
| Cerebral infarction | 1 1 (0.1) |
| Coma | 1 1 (0.1) |
| Epilepsy | 1 1 (0.1) |
| Haemorrhage intracranial | 1 1 (0.1) |
| Seizure | 1 1 (0.1) |
| Speech disorder | 1 1 (0.1) |
| Tremor | 1 1 (0.1) |
| Visual field defect | 1 1 (0.1) |
|  | |
| Blood and lymphatic system disorders | 86 67 (5.8) |
| Blood disorder | 81 64 (5.5) |
| Anaemia | 5 4 (0.3) |
|  | |
| Renal and urinary disorders | 61 56 (4.8) |
| Nephropathy toxic | 57 53 (4.6) |
| Acute kidney injury | 1 1 (0.1) |
| Haematuria | 1 1 (0.1) |
| Proteinuria | 1 1 (0.1) |
| Renal impairment | 1 1 (0.1) |
|  | |
| Metabolism and nutrition disorders | 71 52 ( 4.5) |
| Electrolyte imbalance | 68 49 ( 4.2) |
| Hypokalaemia | 3 3 ( 0.3) |
|  | |
| Gastrointestinal disorders | 57 51 (4.4) |
| Gastrointestinal disorder | 53 48 (4.1) |
| Abdominal pain | 1 1 (0.1) |
| Angular cheilitis | 1 1 (0.1) |
| Gastrointestinal haemorrhage | 1 1 (0.1) |
| Ileus | 1 1 (0.1) |
|  | |
| Ear and labyrinth disorders | 69 32 (2.8) |
| Ototoxicity | 34 31 (2.7) |
| Vestibular disorder | 34 31 (2.7) |
| Tinnitus | 1 1 (0.1) |
|  | |
| Psychiatric disorders | 24 22 (1.9) |
| Psychiatric symptom | 22 20 (1.7) |
| Disorganised speech | 1 1 (0.1) |
| Insomnia | 1 1 (0.1) |
|  | |
| Endocrine disorders | 28 21 (1.8) |
| Thyroid disorder | 28 21 (1.8) |
|  | |
| Respiratory, thoracic and mediastinal disorders | 25 20 (1.7) |
| Haemoptysis | 20 16 (1.4) |
| Pneumothorax | 2 2 (0.2) |
| Dyspnoea | 1 1 (0.1) |
| Respiratory failure | 1 1 (0.1) |
| Tachypnoea | 1 1 (0.1) |
|  | |
| General disorders and administration site conditions | 21 17 (1.5) |
| Death | 10 10 (0.9) |
| Pyrexia | 6 4 (0.3) |
| Chest pain | 3 3 (0.3) |
| Chest discomfort | 2 1 (0.1) |
|  | |
| Skin and subcutaneous tissue disorders | 17 15 (1.3) |
| Rash | 15 13 (1.1) |
| Acne | 2 2 (0.2) |
|  | |
| Cardiac disorders | 16 14 (1.2) |
| Cardiac failure | 6 4 (0.3) |
| Arrhythmia | 3 3 (0.3) |
| Myocardial ischaemia | 2 2 (0.2) |
| Supraventricular tachycardia | 2 2 (0.2) |
| Atrial flutter | 1 1 (0.1) |
| Cardiomyopathy | 1 1 (0.1) |
| Palpitations | 1 1 (0.1) |
|  | |
| Musculoskeletal and connective tissue disorders | 20 11 (0.9) |
| Myalgia | 10 10 (0.9) |
| Arthralgia | 9 9 (0.8) |
| Pain in extremity | 1 1 (0.1) |
|  | |
| Infections and infestations | 7 6 (0.5) |
| Pneumonia | 5 4 (0.3) |
| Upper respiratory tract infection | 2 2 (0.2) |
|  | |
| Eye disorders | 4 4 (0.3) |
| Visual impairment | 3 3 (0.3) |
| Dry eye | 1 1 (0.1) |
|  |  |
| Injury, poisoning and procedural complications | 2 2 (0.2) |
| Overdose | 2 2 (0.2) |
|  | |
| Vascular disorders | 1 1 (0.1) |
| Shock | 1 1 (0.1) |

Note: NE: Number of events.

Percentages are based on the number of all treated subjects.

Adverse events are sorted by descending number of patients. If there are same number of patients in the same SOC or PT, AEs are sorted in alphabet order.

Adverse events are coded using MedDRA, version 22.1.

| Table S2: Summary of ADR by System Organ Class, Preferred Term | |
| --- | --- |
| System Organ Class | Bedaquiline (N=1162) |
| Preferred Term | NE n (%) |
| Number of Subjects with at least one ADR | 516 278 (23.9) |
|  | |
| Investigations | 430 244 (21.0) |
| Electrocardiogram QT prolonged | 427 243 (20.9) |
| Electrocardiogram ST-T change | 1 1 (0.1) |
| Electrocardiogram T wave abnormal | 1 1 (0.1) |
| QRS axis abnormal | 1 1 (0.1) |
|  | |
| Hepatobiliary disorders | 43 34 (2.9) |
| Hepatotoxicity | 43 34 (2.9) |
|  | |
| Gastrointestinal disorders | 16 14 (1.2) |
| Gastrointestinal disorder | 16 14 (1.2) |
|  | |
| Cardiac disorders | 10 10 (0.9) |
| Arrhythmia | 3 3 (0.3) |
| Cardiac failure | 3 3 (0.3) |
| Cardiomyopathy | 1 1 (0.1) |
| Myocardial ischaemia | 1 1 (0.1) |
| Palpitations | 1 1 (0.1) |
| Supraventricular tachycardia | 1 1 (0.1) |
|  | |
| Skin and subcutaneous tissue disorders | 6 5 (0.4) |
| Rash | 4 3 (0.3) |
| Acne | 2 2 (0.2) |
|  | |
| General disorders and administration site conditions | 4 4 (0.3) |
| Pyrexia | 3 3 (0.3) |
| Chest pain | 1 1 (0.1) |
|  | |
| Nervous system disorders | 3 3 (0.3) |
| Headache | 1 1 (0.1) |
| Seizure | 1 1 (0.1) |
| Syncope | 1 1 (0.1) |
|  | |
| Infections and infestations | 1 1 (0.1) |
| Pneumonia | 1 1 (0.1) |
|  | |
| Injury, poisoning and procedural complications | 1 1 (0.1) |
| Overdose | 1 1 (0.1) |
|  | |
| Psychiatric disorders | 1 1 (0.1) |
| Psychiatric symptom | 1 1 (0.1) |
|  | |
| Respiratory, thoracic and mediastinal disorders | 1 1 (0.1) |
| Haemoptysis | 1 1 (0.1) |

Note: NE: Number of events.

Percentages are based on the number of all treated subjects.

Adverse events are sorted by descending number of patients. If there are same number of patients in the same SOC or PT, AEs are sorted in alphabet order.

Adverse events are coded using MedDRA, version 22.1.

If the causality with bedaquiline was missing, the event was defined and summarized as related event but not included in ADR number.

| Table S3: Summary of SAE by System Organ Class, Preferred Term | |
| --- | --- |
| System Organ Class | Bedaquiline (N=1162) |
| Preferred Term | NE n (%) |
| Number of Subjects with at least one SAE | 151 91 ( 7.8) |
|  | |
| Investigations | 83 40 ( 3.4) |
| Electrocardiogram QT prolonged | 83 40 ( 3.4) |
|  | |
| Blood and lymphatic system disorders | 12 12 ( 1.0) |
| Blood disorder | 11 11 ( 0.9) |
| Anaemia | 1 1 ( 0.1) |
|  | |
| General disorders and administration site conditions | 12 12 ( 1.0) |
| Death | 10 10 ( 0.9) |
| Chest pain | 1 1 ( 0.1) |
| Pyrexia | 1 1 ( 0.1) |
|  | |
| Hepatobiliary disorders | 8 7 ( 0.6) |
| Hepatotoxicity | 8 7 ( 0.6) |
|  | |
| Respiratory, thoracic and mediastinal disorders | 8 7 ( 0.6) |
| Haemoptysis | 6 5 ( 0.4) |
| Dyspnoea | 1 1 ( 0.1) |
| Respiratory failure | 1 1 ( 0.1) |
|  | |
| Cardiac disorders | 8 6 ( 0.5) |
| Cardiac failure | 6 4 ( 0.3) |
| Atrial flutter | 1 1 ( 0.1) |
| Cardiomyopathy | 1 1 ( 0.1) |
|  | |
| Nervous system disorders | 7 6 ( 0.5) |
| Neuropathy peripheral | 3 2 ( 0.2) |
| Coma | 1 1 ( 0.1) |
| Epilepsy | 1 1 ( 0.1) |
| Haemorrhage intracranial | 1 1 ( 0.1) |
| Optic neuritis | 1 1 ( 0.1) |
|  | |
| Gastrointestinal disorders | 4 4 ( 0.3) |
| Gastrointestinal disorder | 2 2 ( 0.2) |
| Gastrointestinal haemorrhage | 1 1 ( 0.1) |
| Ileus | 1 1 ( 0.1) |
|  | |
| Infections and infestations | 3 3 ( 0.3) |
| Pneumonia | 3 3 ( 0.3) |
|  | |
| Renal and urinary disorders | 2 2 ( 0.2) |
| Acute kidney injury | 1 1 ( 0.1) |
| Nephropathy toxic | 1 1 ( 0.1) |
|  | |
| Musculoskeletal and connective tissue disorders | 1 1 ( 0.1) |
| Myalgia | 1 1 ( 0.1) |
|  | |
| Psychiatric disorders | 1 1 ( 0.1) |
| Psychiatric symptom | 1 1 ( 0.1) |
|  | |
| Skin and subcutaneous tissue disorders | 1 1 ( 0.1) |
| Rash | 1 1 ( 0.1) |
|  | |
| Vascular disorders | 1 1 ( 0.1) |
| Shock | 1 1 ( 0.1) |

Note: NE: Number of events.

Percentages are based on the number of all treated subjects.

Adverse events are sorted by descending number of patients. If there are same number of patients in the same SOC or PT, AEs are sorted in alphabet order.

Adverse events are coded using MedDRA, version 22.1.

| Table S4: Summary of SADR by System Organ Class and Preferred Term | |
| --- | --- |
| System Organ Class | Bedaquiline (N=1162) |
| Preferred Term | NE n (%) |
| Number of Subjects with at least one SADR | 68 45 ( 3.9) |
|  | |
| Investigations | 56 35 ( 3.0) |
| Electrocardiogram QT prolonged | 56 35 ( 3.0) |
|  | |
| Hepatobiliary disorders | 6 5 ( 0.4) |
| Hepatotoxicity | 6 5 ( 0.4) |
|  | |
| Cardiac disorders | 4 4 ( 0.3) |
| Cardiac failure | 3 3 ( 0.3) |
| Cardiomyopathy | 1 1 ( 0.1) |
|  | |
| Gastrointestinal disorders | 1 1 ( 0.1) |
| Gastrointestinal disorder | 1 1 ( 0.1) |
|  | |
| General disorders and administration site conditions | 1 1 ( 0.1) |
| Pyrexia | 1 1 ( 0.1) |

Note: NE: Number of events.

Percentages are based on the number of all treated subjects.

In the summary outputs, adverse events are sorted by descending number of patients. If there are same number of patients in the same SOC or PT, AEs are sorted in alphabet order.

Adverse events are coded using MedDRA, version 22.1.

Note: If the causality with bedaquiline was missing, the event was defined and summarized as related event but not included in ADR number.

| TableS5: Summary of AE by System Organ Class, Preferred Term and Severity | | | | | |
| --- | --- | --- | --- | --- | --- |
|  | Bedaquiline (N=1162) | | | | |
| System Organ Class  Preferred Term | Grade 1 n (%) | Grade 2 n (%) | Grade 3 n (%) | Grade 4 n (%) | Grade 5 (death) n (%) |
| Total Number of AE | 691 | 354 | 439 | 43 | 14 |
|  | | | | | |
| Number of Subjects with at least one AE | 149 (12.8) | 122 (10.5) | 219 (18.8) | 38 (3.3) | 13 (1.1) |
|  | | | | | |
| General disorders and administration site conditions | 0 | 4 (0.3) | 2 (0.2) | 1 (0.1) | 10 (0.9) |
| Death | 0 | 0 | 0 | 0 | 10 (0.9) |
| Chest discomfort | 0 | 1 (0.1) | 0 | 0 | 0 |
| Chest pain | 0 | 2 (0.2) | 0 | 1 (0.1) | 0 |
| Pyrexia | 1 (0.1) | 1 (0.1) | 2 (0.2) | 0 | 0 |
|  | | | | | |
| Cardiac disorders | 2 (0.2) | 3 (0.3) | 4 (0.3) | 2 (0.2) | 2 (0.2) |
| Cardiac failure | 0 | 0 | 1 (0.1) | 1 (0.1) | 2 (0.2) |
| Arrhythmia | 1 (0.1) | 1 (0.1) | 1 (0.1) | 0 | 0 |
| Cardiomyopathy | 0 | 0 | 1 (0.1) | 0 | 0 |
| Myocardial ischaemia | 1 ( 0.1) | 1 ( 0.1) | 0 | 0 | 0 |
| Palpitations | 0 | 1 ( 0.1) | 0 | 0 | 0 |
| Supraventricular tachycardia | 0 | 0 | 1 ( 0.1) | 1 ( 0.1) | 0 |
|  |  |  |  |  |  |
| Infections and infestations | 1 ( 0.1) | 2 ( 0.2) | 0 | 2 ( 0.2) | 1 ( 0.1) |
| Pneumonia | 0 | 1 ( 0.1) | 0 | 2 ( 0.2) | 1 ( 0.1) |
| Upper respiratory tract infection | 1 ( 0.1) | 1 ( 0.1) | 0 | 0 | 0 |
|  | | | | | |
| Respiratory, thoracic and mediastinal disorders | 11 ( 0.9) | 0 | 6 ( 0.5) | 2 ( 0.2) | 1 ( 0.1) |
| Respiratory failure | 0 | 0 | 0 | 0 | 1 ( 0.1) |
| Dyspnoea | 0 | 0 | 1 ( 0.1) | 0 | 0 |
| Haemoptysis | 10 ( 0.9) | 1 ( 0.1) | 3 ( 0.3) | 2 ( 0.2) | 0 |
| Pneumothorax | 1 ( 0.1) | 0 | 1 ( 0.1) | 0 | 0 |
| Tachypnoea | 0 | 0 | 1 ( 0.1) | 0 | 0 |
|  | | | | | |
| Blood and lymphatic system disorders | 14 ( 1.2) | 11 ( 0.9) | 25 ( 2.2) | 16 ( 1.4) | 0 |
| Anaemia | 0 | 0 | 3 ( 0.3) | 1 ( 0.1) | 0 |
| Blood disorder | 14 ( 1.2) | 12 ( 1.0) | 22 ( 1.9) | 15 ( 1.3) | 0 |
|  | | | | | |
| Ear and labyrinth disorders | 19 ( 1.6) | 11 ( 0.9) | 2 ( 0.2) | 0 | 0 |
| Ototoxicity | 18 ( 1.5) | 11 ( 0.9) | 2 ( 0.2) | 0 | 0 |
| Tinnitus | 1 ( 0.1) | 0 | 0 | 0 | 0 |
| Vestibular disorder | 18 ( 1.5) | 11 ( 0.9) | 2 ( 0.2) | 0 | 0 |
|  |  |  |  |  |  |
| Endocrine disorders | 14 ( 1.2) | 6 ( 0.5) | 0 | 0 | 0 |
| Thyroid disorder | 14 ( 1.2) | 6 ( 0.5) | 0 | 0 | 0 |
|  | | | | | |
| Eye disorders | 2 ( 0.2) | 2 ( 0.2) | 0 | 0 | 0 |
| Dry eye | 1 ( 0.1) | 0 | 0 | 0 | 0 |
| Visual impairment | 1 ( 0.1) | 2 ( 0.2) | 0 | 0 | 0 |
|  | | | | | |
| Gastrointestinal disorders | 23 ( 2.0) | 19 ( 1.6) | 5 ( 0.4) | 3 ( 0.3) | 0 |
| Abdominal pain | 1 ( 0.1) | 0 | 0 | 0 | 0 |
| Angular cheilitis | 1 ( 0.1) | 0 | 0 | 0 | 0 |
| Gastrointestinal disorder | 22 ( 1.9) | 19 ( 1.6) | 4 ( 0.3) | 3 ( 0.3) | 0 |
| Ileus | 0 | 0 | 1 ( 0.1) | 0 | 0 |
|  | | | | | |
| Hepatobiliary disorders | 132 (11.4) | 43 ( 3.7) | 10 ( 0.9) | 5 ( 0.4) | 0 |
| Hepatic function abnormal | 1 ( 0.1) | 0 | 0 | 0 | 0 |
| Hepatotoxicity | 130 (11.2) | 43 ( 3.7) | 10 ( 0.9) | 5 ( 0.4) | 0 |
| Hyperbilirubinaemia | 1 ( 0.1) | 0 | 0 | 0 | 0 |
|  |  |  |  |  |  |
| Injury, poisoning and procedural complications | 1 ( 0.1) | 0 | 1 ( 0.1) | 0 | 0 |
| Overdose | 1 ( 0.1) | 0 | 1 ( 0.1) | 0 | 0 |
|  | | | | | |
| Investigations | 49 ( 4.2) | 52 ( 4.5) | 184 (15.8) | 4 ( 0.3) | 0 |
| Blood urine present | 1 ( 0.1) | 0 | 0 | 0 | 0 |
| Electrocardiogram QT prolonged | 49 ( 4.2) | 51 ( 4.4) | 182 (15.7) | 4 ( 0.3) | 0 |
| Electrocardiogram ST-T change | 0 | 1 ( 0.1) | 0 | 0 | 0 |
| Electrocardiogram T wave abnormal | 0 | 1 ( 0.1) | 0 | 0 | 0 |
| Heart rate decreased | 0 | 0 | 2 ( 0.2) | 0 | 0 |
| Platelet count decreased | 0 | 1 ( 0.1) | 0 | 0 | 0 |
| QRS axis abnormal | 0 | 1 ( 0.1) | 0 | 0 | 0 |
|  | | | | | |
| Metabolism and nutrition disorders | 38 ( 3.3) | 12 ( 1.0) | 0 | 2 ( 0.2) | 0 |
| Electrolyte imbalance | 36 ( 3.1) | 11 ( 0.9) | 0 | 2 ( 0.2) | 0 |
| Hypokalaemia | 2 ( 0.2) | 1 ( 0.1) | 0 | 0 | 0 |
|  | | | | | |
| Musculoskeletal and connective tissue disorders | 2 ( 0.2) | 6 ( 0.5) | 2 ( 0.2) | 0 | 0 |
| Arthralgia | 1 ( 0.1) | 6 ( 0.5) | 1 ( 0.1) | 0 | 0 |
| Myalgia | 1 ( 0.1) | 6 ( 0.5) | 2 ( 0.2) | 0 | 0 |
| Pain in extremity | 1 ( 0.1) | 0 | 0 | 0 | 0 |
|  |  |  |  |  |  |
| Nervous system disorders | 35 ( 3.0) | 43 ( 3.7) | 11 ( 0.9) | 4 ( 0.3) | 0 |
| Cerebral infarction | 0 | 1 ( 0.1) | 0 | 0 | 0 |
| Coma | 0 | 0 | 0 | 1 ( 0.1) | 0 |
| Dizziness | 3 ( 0.3) | 2 ( 0.2) | 0 | 0 | 0 |
| Epilepsy | 0 | 0 | 0 | 1 ( 0.1) | 0 |
| Haemorrhage intracranial | 0 | 0 | 0 | 1 ( 0.1) | 0 |
| Headache | 4 ( 0.3) | 2 ( 0.2) | 0 | 0 | 0 |
| Hypoaesthesia | 0 | 2 ( 0.2) | 0 | 0 | 0 |
| Neuropathy peripheral | 16 ( 1.4) | 22 ( 1.9) | 9 ( 0.8) | 0 | 0 |
| Optic neuritis | 10 ( 0.9) | 16 ( 1.4) | 1 ( 0.1) | 1 ( 0.1) | 0 |
| Seizure | 0 | 0 | 1 ( 0.1) | 0 | 0 |
| Speech disorder | 1 ( 0.1) | 0 | 0 | 0 | 0 |
| Syncope | 1 ( 0.1) | 2 ( 0.2) | 0 | 0 | 0 |
| Tremor | 1 ( 0.1) | 0 | 0 | 0 | 0 |
|  | | | | | |
| Psychiatric disorders | 8 ( 0.7) | 11 ( 0.9) | 2 ( 0.2) | 0 | 0 |
| Disorganised speech | 1 ( 0.1) | 0 | 0 | 0 | 0 |
| Insomnia | 0 | 1 ( 0.1) | 0 | 0 | 0 |
| Psychiatric symptom | 7 ( 0.6) | 10 ( 0.9) | 2 ( 0.2) | 0 | 0 |
|  | | | | | |
| Renal and urinary disorders | 33 ( 2.8) | 17 ( 1.5) | 4 ( 0.3) | 0 | 0 |
| Haematuria | 1 ( 0.1) | 0 | 0 | 0 | 0 |
| Nephropathy toxic | 32 ( 2.8) | 17 ( 1.5) | 3 ( 0.3) | 0 | 0 |
| Proteinuria | 1 ( 0.1) | 0 | 0 | 0 | 0 |
| Renal impairment | 0 | 0 | 1 ( 0.1) | 0 | 0 |
|  | | | | | |
| Skin and subcutaneous tissue disorders | 8 ( 0.7) | 4 ( 0.3) | 3 ( 0.3) | 0 | 0 |
| Acne | 2 ( 0.2) | 0 | 0 | 0 | 0 |
| Rash | 6 ( 0.5) | 4 ( 0.3) | 3 ( 0.3) | 0 | 0 |

Note: NE: Number of events.

Percentages are based on the number of all treated subjects.

Adverse events are sorted by descending number of patients. If there are same number of patients in the same SOC or PT, AEs are sorted in alphabet order.

Adverse events are coded using MedDRA, version 22.1.

| Table S6: Summary of ADR by System Organ Class, Preferred Term and Severity | | | | | |
| --- | --- | --- | --- | --- | --- |
|  | Bedaquiline (N=1162) | | | | |
| System Organ Class  Preferred Term | Grade 1 n (%) | Grade 2 n (%) | Grade 3 n (%) | Grade 4 n (%) | Grade 5 (death) n (%) |
| Total Number of ADR | 147 | 114 | 241 | 14 | 0 |
|  | | | | | |
| Number of Subjects with at least one ADR | 63 ( 5.4) | 55 ( 4.7) | 148 (12.7) | 12 ( 1.0) | 0 |
|  | | | | | |
| Cardiac disorders | 1 ( 0.1) | 3 ( 0.3) | 4 ( 0.3) | 2 ( 0.2) | 0 |
| Arrhythmia | 1 ( 0.1) | 1 ( 0.1) | 1 ( 0.1) | 0 | 0 |
| Cardiac failure | 0 | 0 | 2 ( 0.2) | 1 ( 0.1) | 0 |
| Cardiomyopathy | 0 | 0 | 1 ( 0.1) | 0 | 0 |
| Myocardial ischaemia | 0 | 1 ( 0.1) | 0 | 0 | 0 |
| Palpitations | 0 | 1 ( 0.1) | 0 | 0 | 0 |
| Supraventricular tachycardia | 0 | 0 | 0 | 1 ( 0.1) | 0 |
|  | | | | | |
| Gastrointestinal disorders | 10 ( 0.9) | 3 ( 0.3) | 0 | 1 ( 0.1) | 0 |
| Gastrointestinal disorder | 10 ( 0.9) | 3 ( 0.3) | 0 | 1 ( 0.1) | 0 |
|  | | | | | |
| General disorders and administration site conditions | 0 | 3 ( 0.3) | 1 ( 0.1) | 0 | 0 |
| Chest pain | 0 | 1 ( 0.1) | 0 | 0 | 0 |
| Pyrexia | 0 | 2 ( 0.2) | 1 ( 0.1) | 0 | 0 |
|  | | | | | |
| Hepatobiliary disorders | 22 ( 1.9) | 6 ( 0.5) | 1 ( 0.1) | 5 ( 0.4) | 0 |
| Hepatotoxicity | 22 ( 1.9) | 6 ( 0.5) | 1 ( 0.1) | 5 ( 0.4) | 0 |
|  | | | | | |
| Infections and infestations | 0 | 1 ( 0.1) | 0 | 0 | 0 |
| Pneumonia | 0 | 1 ( 0.1) | 0 | 0 | 0 |
|  |  |  |  |  |  |
| Injury, poisoning and procedural complications | 0 | 0 | 1 ( 0.1) | 0 | 0 |
| Overdose | 0 | 0 | 1 ( 0.1) | 0 | 0 |
|  | | | | | |
| Investigations | 46 ( 4.0) | 50 ( 4.3) | 144 (12.4) | 4 ( 0.3) | 0 |
| Electrocardiogram QT prolonged | 47 ( 4.0) | 48 ( 4.1) | 144 (12.4) | 4 ( 0.3) | 0 |
| Electrocardiogram ST-T change | 0 | 1 ( 0.1) | 0 | 0 | 0 |
| Electrocardiogram T wave abnormal | 0 | 1 ( 0.1) | 0 | 0 | 0 |
| QRS axis abnormal | 0 | 1 ( 0.1) | 0 | 0 | 0 |
|  | | | | | |
| Nervous system disorders | 2 ( 0.2) | 0 | 1 ( 0.1) | 0 | 0 |
| Headache | 1 ( 0.1) | 0 | 0 | 0 | 0 |
| Seizure | 0 | 0 | 1 ( 0.1) | 0 | 0 |
| Syncope | 1 ( 0.1) | 0 | 0 | 0 | 0 |
|  | | | | | |
| Psychiatric disorders | 0 | 1 ( 0.1) | 0 | 0 | 0 |
| Psychiatric symptom | 0 | 1 ( 0.1) | 0 | 0 | 0 |
|  | | | | | |
| Respiratory, thoracic and mediastinal disorders | 1 ( 0.1) | 0 | 0 | 0 | 0 |
| Haemoptysis | 1 ( 0.1) | 0 | 0 | 0 | 0 |
|  | | | | | |
| Skin and subcutaneous tissue disorders | 2 ( 0.2) | 3 ( 0.3) | 0 | 0 | 0 |
| Acne | 2 ( 0.2) | 0 | 0 | 0 | 0 |
| Rash | 0 | 3 ( 0.3) | 0 | 0 | 0 |

Note: NE: Number of events.

Percentages are based on the number of all treated subjects.

In the summary outputs, adverse events are sorted by descending number of patients. If there are same number of patients in the same SOC or PT, AEs are sorted in alphabet order.

Adverse events are coded using MedDRA, version 22.1.

Note: If the causality with bedaquiline was missing, the event was defined and summarized as related event but not included in ADR number.

| Table S7: Summary of AE by System Organ Class, Preferred Term and Outcomes | | | | | | | |
| --- | --- | --- | --- | --- | --- | --- | --- |
|  | Bedaquiline (N=1162) | | | | | | |
| System Organ Class  Preferred Term | Unknown n (%) | Cured n (%) | Improvement n (%) | No Improvement n (%) | | With Sequelae n (%) | Death n (%) |
| Total Number of AE | 194 | 373 | 632 | | 178 | 10 | 18 |
|  | | | | | | | |
| Number of Subjects with at least one AE | 41 ( 3.5) | 78 ( 6.7) | 263 (22.6) | | 95 ( 8.2) | 5 ( 0.4) | 14 ( 1.2) |
|  | | | | | | | |
| General disorders and administration site conditions | 2 ( 0.2) | 2 ( 0.2) | 3 ( 0.3) | | 0 | 0 | 10 ( 0.9) |
| Death | 0 | 0 | 0 | | 0 | 0 | 10 ( 0.9) |
| Chest discomfort | 0 | 0 | 1 ( 0.1) | | 0 | 0 | 0 |
| Chest pain | 2 ( 0.2) | 0 | 1 ( 0.1) | | 0 | 0 | 0 |
| Pyrexia | 0 | 3 ( 0.3) | 1 ( 0.1) | | 0 | 0 | 0 |
|  | | | | | | | |
| Cardiac disorders | 1 ( 0.1) | 5 ( 0.4) | 5 ( 0.4) | | 0 | 0 | 2 ( 0.2) |
| Cardiac failure | 1 ( 0.1) | 1 ( 0.1) | 0 | | 0 | 0 | 2 ( 0.2) |
| Arrhythmia | 0 | 1 ( 0.1) | 2 ( 0.2) | | 0 | 0 | 0 |
| Atrial flutter | 0 | 1 ( 0.1) | 0 | | 0 | 0 | 0 |
| Myocardial ischaemia | 0 | 1 ( 0.1) | 1 ( 0.1) | | 0 | 0 | 0 |
| Palpitations | 0 | 0 | 1 ( 0.1) | | 0 | 0 | 0 |
| Supraventricular tachycardia | 0 | 1 ( 0.1) | 1 ( 0.1) | | 0 | 0 | 0 |
|  | | | | | | | |
| Gastrointestinal disorders | 7 ( 0.6) | 8 ( 0.7) | 27 ( 2.3) | | 4 ( 0.3) | 0 | 1 ( 0.1) |
| Gastrointestinal haemorrhage | 0 | 0 | 0 | | 0 | 0 | 1 ( 0.1) |
| Abdominal pain | 0 | 0 | 1 ( 0.1) | | 0 | 0 | 0 |
| Angular cheilitis | 0 | 1 ( 0.1) | 0 | | 0 | 0 | 0 |
| Gastrointestinal disorder | 7 ( 0.6) | 7 ( 0.6) | 26 ( 2.2) | | 4 ( 0.3) | 0 | 0 |
| Ileus | 0 | 1 ( 0.1) | 0 | | 0 | 0 | 0 |
|  | | | | | | | |
| Hepatobiliary disorders | 20 ( 1.7) | 41 ( 3.5) | 89 ( 7.7) | | 20 ( 1.7) | 1 ( 0.1) | 1 ( 0.1) |
| Hepatotoxicity | 20 ( 1.7) | 41 ( 3.5) | 88 ( 7.6) | | 20 ( 1.7) | 1 ( 0.1) | 1 ( 0.1) |
| Hepatic function abnormal | 0 | 0 | 0 | | 1 ( 0.1) | 0 | 0 |
| Hyperbilirubinaemia | 0 | 0 | 1 ( 0.1) | | 0 | 0 | 0 |
|  |  |  |  | |  |  |  |
| Infections and infestations | 0 | 1 ( 0.1) | 3 ( 0.3) | | 1 ( 0.1) | 0 | 1 ( 0.1) |
| Pneumonia | 0 | 1 ( 0.1) | 1 ( 0.1) | | 1 ( 0.1) | 0 | 1 ( 0.1) |
| Upper respiratory tract infection | 0 | 0 | 2 ( 0.2) | | 0 | 0 | 0 |
|  | | | | | | | |
| Renal and urinary disorders | 8 ( 0.7) | 10 ( 0.9) | 21 ( 1.8) | | 8 ( 0.7) | 0 | 1 ( 0.1) |
| Acute kidney injury | 0 | 0 | 0 | | 0 | 0 | 1 ( 0.1) |
| Haematuria | 0 | 0 | 1 ( 0.1) | | 0 | 0 | 0 |
| Nephropathy toxic | 8 ( 0.7) | 10 ( 0.9) | 20 ( 1.7) | | 7 ( 0.6) | 0 | 0 |
| Proteinuria | 0 | 0 | 1 ( 0.1) | | 0 | 0 | 0 |
| Renal impairment | 0 | 0 | 0 | | 1 ( 0.1) | 0 | 0 |
|  | | | | | | | |
| Respiratory, thoracic and mediastinal disorders | 1 ( 0.1) | 6 ( 0.5) | 9 ( 0.8) | | 1 ( 0.1) | 0 | 1 ( 0.1) |
| Respiratory failure | 0 | 0 | 0 | | 0 | 0 | 1 ( 0.1) |
| Dyspnoea | 0 | 0 | 1 ( 0.1) | | 0 | 0 | 0 |
| Haemoptysis | 1 ( 0.1) | 6 ( 0.5) | 7 ( 0.6) | | 1 ( 0.1) | 0 | 0 |
| Pneumothorax | 0 | 0 | 1 ( 0.1) | | 0 | 0 | 0 |
| Tachypnoea | 0 | 0 | 1 ( 0.1) | | 0 | 0 | 0 |
|  | | | | | | | |
| Vascular disorders | 0 | 0 | 0 | | 0 | 0 | 1 ( 0.1) |
| Shock | 0 | 0 | 0 | | 0 | 0 | 1 ( 0.1) |
|  | | | | | | | |
| Blood and lymphatic system disorders | 4 ( 0.3) | 13 ( 1.1) | 34 ( 2.9) | | 11 ( 0.9) | 1 ( 0.1) | 0 |
| Anaemia | 0 | 2 ( 0.2) | 2 ( 0.2) | | 0 | 0 | 0 |
| Blood disorder | 4 ( 0.3) | 11 ( 0.9) | 33 ( 2.8) | | 11 ( 0.9) | 1 ( 0.1) | 0 |
|  |  |  |  | |  |  |  |
| Ear and labyrinth disorders | 1 ( 0.1) | 5 ( 0.4) | 17 ( 1.5) | | 5 ( 0.4) | 2 ( 0.2) | 0 |
| Ototoxicity | 1 ( 0.1) | 5 ( 0.4) | 16 ( 1.4) | | 5 ( 0.4) | 2 ( 0.2) | 0 |
| Tinnitus | 0 | 0 | 1 ( 0.1) | | 0 | 0 | 0 |
| Vestibular disorder | 1 ( 0.1) | 5 ( 0.4) | 16 ( 1.4) | | 5 ( 0.4) | 2 ( 0.2) | 0 |
|  | | | | | | | |
| Endocrine disorders | 1 ( 0.1) | 2 ( 0.2) | 8 ( 0.7) | | 6 ( 0.5) | 0 | 0 |
| Thyroid disorder | 1 ( 0.1) | 2 ( 0.2) | 8 ( 0.7) | | 6 ( 0.5) | 0 | 0 |
|  | | | | | | | |
| Eye disorders | 0 | 0 | 1 ( 0.1) | | 3 ( 0.3) | 0 | 0 |
| Dry eye | 0 | 0 | 1 ( 0.1) | | 0 | 0 | 0 |
| Visual impairment | 0 | 0 | 0 | | 3 ( 0.3) | 0 | 0 |
|  |  |  |  | |  |  |  |
| Injury, poisoning and procedural complications | 1 ( 0.1) | 0 | 1 ( 0.1) | | 0 | 0 | 0 |
| Overdose | 1 ( 0.1) | 0 | 1 ( 0.1) | | 0 | 0 | 0 |
|  | | | | | | | |
| Investigations | 37 ( 3.2) | 68 ( 5.9) | 123 (10.6) | | 32 ( 2.8) | 0 | 0 |
| Blood urine present | 0 | 1 ( 0.1) | 0 | | 0 | 0 | 0 |
| Electrocardiogram QT prolonged | 37 ( 3.2) | 66 ( 5.7) | 122 (10.5) | | 32 ( 2.8) | 0 | 0 |
| Electrocardiogram ST-T change | 0 | 1 ( 0.1) | 0 | | 0 | 0 | 0 |
| Electrocardiogram T wave abnormal | 0 | 1 ( 0.1) | 0 | | 0 | 0 | 0 |
| Heart rate decreased | 0 | 1 ( 0.1) | 0 | | 0 | 0 | 0 |
| Platelet count decreased | 0 | 0 | 1 ( 0.1) | | 0 | 0 | 0 |
| QRS axis abnormal | 0 | 0 | 1 ( 0.1) | | 0 | 0 | 0 |
|  | | | | | | | |
| Metabolism and nutrition disorders | 8 ( 0.7) | 28 ( 2.4) | 11 ( 0.9) | | 2 ( 0.2) | 0 | 0 |
| Electrolyte imbalance | 8 ( 0.7) | 27 ( 2.3) | 9 ( 0.8) | | 2 ( 0.2) | 0 | 0 |
| Hypokalaemia | 0 | 1 ( 0.1) | 2 ( 0.2) | | 0 | 0 | 0 |
|  | | | | | | | |
| Musculoskeletal and connective tissue disorders | 0 | 1 ( 0.1) | 6 ( 0.5) | | 1 ( 0.1) | 0 | 0 |
| Arthralgia | 0 | 1 ( 0.1) | 5 ( 0.4) | | 0 | 0 | 0 |
| Myalgia | 0 | 1 ( 0.1) | 5 ( 0.4) | | 1 ( 0.1) | 0 | 0 |
| Pain in extremity | 0 | 0 | 1 ( 0.1) | | 0 | 0 | 0 |
|  |  |  |  | |  |  |  |
| Nervous system disorders | 11 ( 0.9) | 7 ( 0.6) | 48 ( 4.1) | | 13 ( 1.1) | 3 ( 0.3) | 0 |
| Cerebral infarction | 0 | 0 | 0 | | 0 | 1 ( 0.1) | 0 |
| Coma | 0 | 0 | 0 | | 1 ( 0.1) | 0 | 0 |
| Dizziness | 0 | 1 ( 0.1) | 4 ( 0.3) | | 0 | 0 | 0 |
| Epilepsy | 0 | 0 | 1 ( 0.1) | | 0 | 0 | 0 |
| Haemorrhage intracranial | 0 | 1 ( 0.1) | 0 | | 0 | 0 | 0 |
| Headache | 1 ( 0.1) | 1 ( 0.1) | 4 ( 0.3) | | 0 | 0 | 0 |
| Hypoaesthesia | 0 | 0 | 1 ( 0.1) | | 0 | 0 | 0 |
| Neuropathy peripheral | 8 ( 0.7) | 1 ( 0.1) | 22 ( 1.9) | | 9 ( 0.8) | 2 ( 0.2) | 0 |
| Optic neuritis | 2 ( 0.2) | 2 ( 0.2) | 15 ( 1.3) | | 2 ( 0.2) | 0 | 0 |
| Seizure | 0 | 0 | 1 ( 0.1) | | 0 | 0 | 0 |
| Speech disorder | 0 | 0 | 1 ( 0.1) | | 0 | 0 | 0 |
| Syncope | 0 | 1 ( 0.1) | 2 ( 0.2) | | 0 | 0 | 0 |
| Tremor | 0 | 1 ( 0.1) | 0 | | 0 | 0 | 0 |
| Visual field defect | 0 | 0 | 0 | | 1 ( 0.1) | 0 | 0 |
|  | | | | | | | |
| Psychiatric disorders | 1 ( 0.1) | 6 ( 0.5) | 14 ( 1.2) | | 1 ( 0.1) | 0 | 0 |
| Disorganised speech | 0 | 0 | 1 ( 0.1) | | 0 | 0 | 0 |
| Insomnia | 0 | 0 | 0 | | 1 ( 0.1) | 0 | 0 |
| Psychiatric symptom | 1 ( 0.1) | 6 ( 0.5) | 13 ( 1.1) | | 0 | 0 | 0 |
|  | | | | | | | |
| Skin and subcutaneous tissue disorders | 0 | 4 ( 0.3) | 5 ( 0.4) | | 6 ( 0.5) | 0 | 0 |
| Acne | 0 | 0 | 0 | | 2 ( 0.2) | 0 | 0 |
| Rash | 0 | 4 ( 0.3) | 5 ( 0.4) | | 4 ( 0.3) | 0 | 0 |

| Table S8: Summary of ADR by System Organ Class, Preferred Term and Outcomes | | | | | | |
| --- | --- | --- | --- | --- | --- | --- |
|  | Bedaquiline (N=1162) | | | | | |
| System Organ Class  Preferred Term | Unknown n (%) | Cured n (%) | Improvement n (%) | No Improvement n (%) | With Sequelae n (%) | Death n (%) |
| Total Number of ADR | 89 | 126 | 201 | 56 | 0 | 0 |
|  | | | | | | |
| Number of Subjects with at least one ADR | 33 ( 2.8) | 67 ( 5.8) | 119 (10.2) | 34 ( 2.9) | 0 | 0 |
|  | | | | | | |
| Cardiac disorders | 1 ( 0.1) | 3 ( 0.3) | 5 ( 0.4) | 0 | 0 | 0 |
| Arrhythmia | 0 | 1 ( 0.1) | 2 ( 0.2) | 0 | 0 | 0 |
| Cardiac failure | 1 ( 0.1) | 1 ( 0.1) | 1 ( 0.1) | 0 | 0 | 0 |
| Myocardial ischaemia | 0 | 0 | 1 ( 0.1) | 0 | 0 | 0 |
| Palpitations | 0 | 0 | 1 ( 0.1) | 0 | 0 | 0 |
| Supraventricular tachycardia | 0 | 1 ( 0.1) | 0 | 0 | 0 | 0 |
|  | | | | | | |
| Gastrointestinal disorders | 3 ( 0.3) | 1 ( 0.1) | 7 ( 0.6) | 1 ( 0.1) | 0 | 0 |
| Gastrointestinal disorder | 3 ( 0.3) | 1 ( 0.1) | 7 ( 0.6) | 1 ( 0.1) | 0 | 0 |
|  | | | | | | |
| General disorders and administration site conditions | 1 ( 0.1) | 2 ( 0.2) | 1 ( 0.1) | 0 | 0 | 0 |
| Chest pain | 1 ( 0.1) | 0 | 0 | 0 | 0 | 0 |
| Pyrexia | 0 | 2 ( 0.2) | 1 ( 0.1) | 0 | 0 | 0 |
|  | | | | | | |
| Hepatobiliary disorders | 7 ( 0.6) | 3 ( 0.3) | 15 ( 1.3) | 6 ( 0.5) | 0 | 0 |
| Hepatotoxicity | 7 ( 0.6) | 3 ( 0.3) | 15 ( 1.3) | 6 ( 0.5) | 0 | 0 |
|  | | | | | | |
| Infections and infestations | 0 | 0 | 1 ( 0.1) | 0 | 0 | 0 |
| Pneumonia | 0 | 0 | 1 ( 0.1) | 0 | 0 | 0 |
|  |  |  |  |  |  |  |
| Injury, poisoning and procedural complications | 1 ( 0.1) | 0 | 0 | 0 | 0 | 0 |
| Overdose | 1 ( 0.1) | 0 | 0 | 0 | 0 | 0 |
|  | | | | | | |
| Investigations | 31 ( 2.7) | 66 ( 5.7) | 100 ( 8.6) | 26 ( 2.2) | 0 | 0 |
| Electrocardiogram QT prolonged | 31 ( 2.7) | 66 ( 5.7) | 99 ( 8.5) | 26 ( 2.2) | 0 | 0 |
| Electrocardiogram ST-T change | 0 | 1 ( 0.1) | 0 | 0 | 0 | 0 |
| Electrocardiogram T wave abnormal | 0 | 1 ( 0.1) | 0 | 0 | 0 | 0 |
| QRS axis abnormal | 0 | 0 | 1 ( 0.1) | 0 | 0 | 0 |
|  | | | | | | |
| Nervous system disorders | 0 | 2 ( 0.2) | 1 ( 0.1) | 0 | 0 | 0 |
| Headache | 0 | 1 ( 0.1) | 0 | 0 | 0 | 0 |
| Seizure | 0 | 0 | 1 ( 0.1) | 0 | 0 | 0 |
| Syncope | 0 | 1 ( 0.1) | 0 | 0 | 0 | 0 |
|  | | | | | | |
| Psychiatric disorders | 0 | 0 | 1 ( 0.1) | 0 | 0 | 0 |
| Psychiatric symptom | 0 | 0 | 1 ( 0.1) | 0 | 0 | 0 |
|  | | | | | | |
| Respiratory, thoracic and mediastinal disorders | 0 | 0 | 1 ( 0.1) | 0 | 0 | 0 |
| Haemoptysis | 0 | 0 | 1 ( 0.1) | 0 | 0 | 0 |
|  | | | | | | |
| Skin and subcutaneous tissue disorders | 1 ( 0.1) | 1 ( 0.1) | 0 | 3 ( 0.3) | 0 | 0 |
| Acne | 0 | 0 | 0 | 2 ( 0.2) | 0 | 0 |
| Rash | 1 ( 0.1) | 1 ( 0.1) | 0 | 1 ( 0.1) | 0 | 0 |

| Table S9: Summary of AE Leading to Death by System Organ Class and Preferred Term | |
| --- | --- |
| System Organ Class | Bedaquiline (N=1162) |
| Preferred Term | NE n (%) |
| Number of Subjects with at least one AE leading to death [1] | 18 14 ( 1.2) |
|  | |
| General disorders and administration site conditions | 10 10 ( 0.9) |
| Death | 10 10 ( 0.9) |
|  | |
| Cardiac disorders | 2 2 ( 0.2) |
| Cardiac failure | 2 2 ( 0.2) |
|  | |
| Gastrointestinal disorders | 1 1 ( 0.1) |
| Gastrointestinal haemorrhage | 1 1 ( 0.1) |
|  | |
| Hepatobiliary disorders | 1 1 ( 0.1) |
| Hepatotoxicity | 1 1 ( 0.1) |
|  | |
| Infections and infestations | 1 1 ( 0.1) |
| Pneumonia | 1 1 ( 0.1) |
|  | |
| Renal and urinary disorders | 1 1 ( 0.1) |
| Acute kidney injury | 1 1 ( 0.1) |
|  | |
| Respiratory, thoracic and mediastinal disorders | 1 1 ( 0.1) |
| Respiratory failure | 1 1 ( 0.1) |
|  | |
| Vascular disorders | 1 1 ( 0.1) |
| Shock | 1 1 ( 0.1) |
